# The Molecular Epidemiology of *Echinococcus* Infections

**DOI:** 10.3390/pathogens9060453

**Published:** 2020-06-08

**Authors:** R. C. Andrew Thompson

**Affiliations:** School of Veterinary and Life Sciences, Murdoch University, Murdoch, WA 6150, Australia; a.thompson@murdoch.edu.au

**Keywords:** *Echinococcus*, taxonomy, molecular epidemiology

## Abstract

Molecular epidemiology (ME) is the application of molecular tools to determine the causation of disease. With infectious diseases, such as echinococcosis, this applies to identifying and characterising the aetiological agents and elucidating host range. Such an approach has been very successful with the causative agents of echinococcosis, species of *Echinococcus*, initially by providing a workable and practical taxonomy and subsequently determining transmission patterns in endemic areas. This review summarises the taxonomy and nomenclature of species of *Echinococcus* and provides an update on ME investigations of the ecology of *Echinococcus* transmission, particularly in areas where more than one species of *Echinococcus* is maintained in cycles of transmission that may interact.

## 1. Molecular Epidemiology

Molecular epidemiology (ME) has revolutionised our understanding of infectious disease transmission. It provides laboratory and analytical tools that can characterise infectious agents in investigations focused on the aetiology of infectious diseases. ME is therefore a powerful resource in both outbreak situations as well as ongoing surveillance activities, particularly with investigations of emerging infectious diseases. ME plays an important role in both human and domestic animal health, and increasingly with infectious agents of wildlife in the context of disease emergence and conservation [1].

## 2. Echinococcosis

Echinococcosis, a global disease of which there are two forms, cystic and alveolar, is caused by the cestode parasite *Echinococcus*. When considering the epidemiology of any infectious disease, understanding its aetiology is central. With echinococcosis, this has been problematic in many endemic areas where different host species are involved and there is a need to understand their role in transmission [2]. This is exacerbated if there are thought to be more than one species, or ‘strains’/genotypes, of *Echinococcus* present [2]. Molecular epidemiology has made a major contribution to understanding the extensive genetic and phenotypic variation exhibited within the genus *Echinococcus* [2,3]. This has resulted in the establishment of a sound taxonomy (Table 1), supported by biological data, and the development of appropriate molecular tools for epidemiological investigations, and the elucidation of transmission patterns. Molecular tools have principally involved PCR-based approaches and sequencing of mitochondrial and nuclear DNA [4,5].

## 3. Life Cycle and Transmission

*Echinococcus* requires two mammalian hosts to complete its life cycle: a carnivore as a definitive host and a herbivorous or omnivorous intermediate host, which may include humans. It has a wide host range, particularly of intermediate hosts, and host specificity varies between species of *Echinococcus* (Table 1). The situation is complicated when different species occur in the same geographical areas with the possibility of interaction between cycles of transmission. This may occur when the same species of definitive host is susceptible to more than one species of *Echinococcus* which has a range of different intermediate hosts, some of which may be susceptible to more than one species of *Echinococcus*. For example, in the Middle East, dogs can be infected with *E. granulosus* and *E. intermedius*, as can sheep and camels (see below). In addition, the situation is often compounded in areas where there is the possibility of spillover between domestic, rural, or urban cycles of transmission and cycles involving wildlife [1]. Such spillover situations may result from wildlife encroaching into urban or peri-urban areas with transmission to susceptible domestic hosts, resulting in the establishment of domestic cycles. In contrast, in some endemic areas such as Australia, the reverse situation has occurred when domestic cycles have resulted in the establishment of *E. granulosus* in previously uninfected wildlife, macropod marsupials and dingoes [6]. In this case, the resulting sylvatic cycle can operate independently but provides a constant source of infection that can spill over back to domestic hosts.

## 4. Taxonomy and Nomenclature

The formal naming of a species is a prerequisite for effective communication which is particularly important when the species in question are of public health importance and require coordinated control efforts. *Echinococcus* has a long history of taxonomic and nomenclatural confusion, particularly regarding the species level taxonomy [2]. This has resulted from a lack of useful morphological characters, and where the importance of host occurrence was often overshadowed by taxonomic considerations [2]. Thus, the ecology of transmission of *Echinococcus* in areas where multiple host species are infected has only recently been interpreted with confidence.

Fortunately, there is now widespread agreement based on morphological, molecular, and ecological criteria that *Echinococcus* should be split into ten species (Table 1). Molecular approaches have been very valuable in resolving taxonomic issues and have largely confirmed original taxonomic considerations, and importantly, from a practical point of view, the reliability of differential morphological characters.

In this review, the species names in Table 1 will be used rather than genotypes.

### 4.1. Echinococcus granulosus

*Echinococcus granulosus* is the most widespread species causing cystic echinococcosis in humans and has the broadest intermediate host range [2,3]. It is principally maintained in cycles involving livestock but spillover into wildlife occurs in some areas, such as Australia (see above). *E. granulosus* is also common in areas where there are a range of intermediate hosts potentially susceptible to other species, apart from *E. granulosus*. Molecular tools play an important role in such situations allowing species identification from metacestode stages. For example, in parts of Europe and the Middle East, sheep, cattle, horses, pigs, and camels may be infected with *E. granulosus*, *E. equinus*, *E. ortleppi*, and *E. intermedius*, all of which, apart from *E. equinus*, are of public health significance [2,3,7]. In such situations, it is important to determine whether separate species do occur sympatrically, impeding control efforts and focussed public health interventions. For example, in many areas of the Middle East, *E. granulosus* and *E. intermedius* are both maintained sympatrically in cycles, with sheep and camels as intermediate hosts and dogs as definitive hosts [7]. Humans may be infected with either species sometimes in mixed infections [8]. In Bolivia, a recent ME study reported the presence of *E. granulosus*, *E. ortleppi*, and *E. intermedius* in sheep, cattle, and pigs, respectively [9]. Camels play the dominant role as intermediate host of *E. intermedius* and to a lesser extent, *E. granulosus*, in many Middle Eastern regions. However, in Mazandaran Province in Iran, *E. granulosus* was found in an ME study to be the only species in livestock and humans [10] and in Saudi Arabia, an ME study in Riyadh found only *E. granulosus* in sheep and camels [11]. Cattle have been considered to play a minor role in the transmission of *E. granulosus*. However, recent ME studies in Sudan and Ethiopia demonstrated cattle to be epidemiologically the most important intermediate host for *E. granulosus* as well as *E. ortleppi* and *E. intermedius* [12,13].

In the past, three genotypes were identified within the species *E. granulosus*, G1, G2, and G3, based on phenotypic differences (G2) and host preference (buffaloes, G3) [2]. The taxonomic status of G2 and G3 has been uncertain. However, recent molecular characterisation at various loci [5,14] has provided no evidence that G2 and G3 warrant species delimitation, but genotypic recognition should be retained given the epidemiologically important phenotypic characteristics.

### 4.2. Echinococcus equinus and E. ortleppi

The life cycles of *E. equinus* and *E. ortleppi* both involve dogs as definitive hosts and horses and cattle as intermediate hosts, respectively [2]. Both species exhibit high host specificity for both definitive and intermediate hosts and only *E. ortleppi* is infective to humans. Their high host specificity probably explains why both species do not have a broad geographical distribution and occur sporadically, since access of dogs to the metacestode stages in horses and cattle is infrequent, particularly as public health measures have improved in endemic areas. However, recent ME studies have demonstrated that transmission of both species is still occurring in Turkey [15], Africa, South America, and parts of Europe [16]. Interestingly, the first ME study undertaken on echinococcosis in Bhutan demonstrated local transmission of both *E. ortleppi* and *E. granulosus* [17].

### 4.3. Echinococcus canadensis and E. intermedius

*Echinococcus* maintained in cycles involving wild and farmed cervids, moose (*Alces alces*), elk (wapiti) (*Cervus elephas*), and reindeer (caribou) (*Rangifer tarandus*), domestic pigs, and camels as intermediate hosts has long been recognised as distinct from *E. granulosus* [2]. ME studies initially confirmed that four distinct genotypes of *Echinococcus*, G6, 7, 8, and 10, are maintained in these life cycles [18]. These studies complemented morphological descriptions of the adult parasites of cervid, pig, and camel origin (reviewed in Reference [18]). Although molecular tools have been valuable in confirming the genetic distinctness of these forms, and subsequently, ME studies have demonstrated different host ranges and geographic distributions, nomenclatural issues have impeded a clear understanding of their transmission cycles (reviewed in Reference [18]).

G8 and G10 are variants of the one species *E. canadensis* [2]. Initially, it was proposed that the genotypes G6 and G7 should also be included within the species *E. canadensis* [19]. Unfortunately, this did not reflect their ecology or geographical distribution [18]. Molecular approaches have now confirmed that the four genotypes represent two, possibly three species [18,20]. G6 and G7 are clearly distinct from sequences of wildlife isolates of G8 and G10, and sequences of the latter were remarkably distant from each other [16]. Molecular phylogeny based on six nuclear genes demonstrated that G6/G7 and G8/G10 should be regarded as two distinct species, *E. intermedius* and *E. canadensis* [20] (Table 1). This was recommended earlier [21] since the name *E. intermedius* had been previously proposed for the species in pigs and camels (reviewed in Reference [18]). The origin of the name *E. intermedius* has been discussed in detail and since it emanates from a country, Spain (reviewed in Reference [18]), where *E. granulosus*, *E. equinus*, and G7/*E. intermedius* are maintained sympatrically in separate cycles of transmission involving sheep, hoses, and pigs respectively (reviewed in Reference [18]), retaining the name *E. intermedius* is appropriate for denoting G6/G7 genotypes found in camels, pigs, and other intermediate hosts. Detailed morphological descriptions based on experimental infections for the camel and pig forms are available and complement the molecular data (see Reference [18]).

Both genotypes of *E. canadensis* are principally restricted to their wildlife hosts, wolves and cervids in North America and Scandinavia, and occasionally infect humans [7]. In contrast, infections with *E. intermedius* occur principally in the Middle East, Africa, and Europe, often where *E. granulosus* is also endemic, and are maintained in domestic cycles and are infective to humans [7]. The known geographical distribution of *E. intermedius* is likely to increase as further ME surveillance is undertaken. For example, recent reports from a number of West African countries, including Nigeria, suggest that the genotype G6 of *E. intermedius* is the dominant species due to the large involvement of camels [22] and therefore of greatest risk to public health of CE in the region.

In a study of *E. canadensis* in wild canids in Quebec and Maine [23], the authors emphasised that although both wolves and coyotes were definitive hosts for *E. canadensis*, coyotes are more likely to contaminate urban green spaces and peri-urban environments than wolves.

### 4.4. Echinococcus oligarthra and E. vogeli

Little is known about the geographical distribution and host range of the two sylvatic species in South America, *E. oligarthra* and *E. vogeli.* Recently, however, molecular surveillance in Argentina using mitochondrial and nuclear markers has extended the host range of *E. oligarthra* to include agoutis (*Dasyprocta azarae*), ocelot (*Leopardus pardalis*), and puma (*Puma concolor*) [24], as well as demonstrating the existence of two genetically different populations of *E. oligarthra* maintained and transmitted in sylvatic cycles in Argentina.

Although both *E. oligarthra* and *E. vogeli* have been reported to infect humans [3], their zoonotic significance is unknown. A recent report from Brazil [25] demonstrated that molecular screening of dog faeces is a useful tool for determining the potential public health significance of *E. vogeli* infections in domestic dogs.

### 4.5. Echinococcus multilocularis

Studies over the last decade have demonstrated a great deal of genetic diversity between isolates of *E. multilocularis* from Europe, Asia, North America, and China, resulting in a number of different strains that are referred to in the literature as: European, Asian, Mongolian, and North American [2,3,26]. There is no evidence that any of these genetic variants warrant taxonomic recognition and until recently, there has been a paucity of studies investigating the epidemiological significance of this genetic variation.

There is now growing evidence that the European strain of *E. multilocularis* may be more virulent than indigenous sylvatic strains in North America. Recent ME studies suggest that the European strain of *E. multilocularis* was recently introduced into Canada with domestic dogs or red foxes, followed by establishment in wildlife [7]. ME detection of these European strains coincided with the detection of cases of alveolar hydatid disease in dogs in Canada for the first time. In some cases, European-type strains have been identified from cyst tissue from affected dogs [7]. This suggests that, in contrast with endemic strains of *E. multilocularis* long-established in central North America, European-type strains may be less intermediate host-specific and may have more zoonotic potential. Epidemiologically, many more human cases of *E. multilocularis* are observed in Europe compared to Canada, where only one autochthonous case was reported before 2013 [7].

In China, ME studies suggest that there is more than one genotype of the Asian strain, with genotype 1 possibly being the most pathogenic and infective to humans [27].

The urbanisation of *E. multilocularis* via the red fox has been well documented throughout Europe and Japan [7]. A similar situation has also emerged involving coyotes infected with *E. multilocularis* in urban areas of North America [7]. Like the red fox, the coyote is a successful urban adapter, and is increasingly seen as a public health risk in urban environments in North America. Recent ME studies in Calgary, Canada, found that coyotes were frequently infected with *E. multilocularis* [7,28].

### 4.6. Echinococcus shiquicus

This enigmatic variant of *Echinococcus multilocularis*, which is seemingly restricted to parts of China [7], remains little understood in terms of host range and zoonotic potential. Recent ME studies are beginning to shed light on aspects of host preference. In contrast to previous studies, a recent study indicated that rodent species, rather than pikas, are probably more important natural intermediate hosts of *E. multilocularis* and *E. shiquicus* in the eastern Tibetan Plateau [29].

## 5. Application of Molecular Epidemiology in Endemic Regions

### 5.1. Multiple Transmission Cycles

Molecular epidemiological studies have been instrumental in identifying and describing cycles of transmission involving more than one species of *Echinococcus.* Although morphology can be used to differentiate between adult worms of the different species, this approach is often not practical given the difficulties in recovering adult specimens and the associated public health risks involved. Molecular tools also allow species identification from eggs in faeces. For example, during a faecal survey of *E. granulosus* in wolves and dogs in the Southern Italian Alps, *E. multilocularis* eggs were unexpectedly detected by molecular analysis in four faecal samples from at least two shepherd dogs, and in five wolf faecal samples [30]. Co-infection of *E. ortleppi* and *E. multiloculalris* was also identified in two wolves. Similarly, co-infections of *E. multilocularis* and *E. canadensis* were reported in coyotes and red foxes in Alberta, Canada, using real-time PCR [30]. Another ME study of wild canids on both sides of the Canada–USA border in eastern North America, found wolves and coyotes infected with *E. canadensis*, and discriminated between the G8 and G10 genotypes, finding single and mixed infections of these genotypes in both canid species [23].

It is only recently that ME studies have been undertaken in Africa and they have revealed an interesting and more complicated situation than originally envisaged. Initially, *E. granulosus* was considered to be the most widespread species, with *E. ortleppi* present but with an apparently much more localised distribution. Although infections in lions was known to occur for many years, it is only relatively recently that adult worms from lions were characterised using molecular tools and confirmed to be a distinct species, *E. felidis* [31]. Interestingly, recent ME studies have demonstrated that *E. felidis* is not confined to a sylvatic cycle and has been identified in domestic dogs in Kenya [32]. ME studies have also demonstrated infections with *E. granulosus, E. intermedius*, and *E. ortleppi* in dogs, including some mixed infections [32].

The situation in China is complex, with multiple species of *Echinococcus* maintained in transmission cycles [3,7]. However, ME studies are required to fully document the distribution of the different species, their host range, and the occurrence of sympatric cycles of transmission.

### 5.2. Human Infections and Clinical Management

In endemic regions where multiple species of *Echinococcus* are maintained in sympatric cycles of transmission, and humans are at risk of infection, it is important to determine the species responsible for clinical infections. This will aid clinical interventions and management [32] but also inform and guide public health measures. Molecular diagnostic approaches have been shown to be valuable tools in this respect. For example, authors of a recent study [33] were able to genetically characterise by sequence analysis the causative species of *Echinococcus* from hydatid fluid of patients in northern and western China. One isolate from a patient was *E. multilocularis* and another *E. intermedius*, and the other 22 isolates examined were all *E. granulosus*.

In another recent ME study in south east Iran of 42 echinococcosis patients, 18 were infected with *E. granulosus* and 24 with *E. intermedius* (G6 genotype) [8]. In one patient, a mixed infection of *E. granulosus* in the liver and right lung, and *E. intermedius* (G6) in the left lung, was detected [8]. This study showed a significantly high proportion of CE patients infected with the G6 genotype, particularly in the southern parts of the province [8].

Similar studies in China are starting to reveal a complex diversity of infection patterns in humans infected with *E. multilocularis*. For example, genotypic diversity in alveolar echinococcosis patients was demonstrated, with most infected with the Asian genotype 1 [27]. The authors concluded that their results may be an indication of the relative frequency of the Asian genotype 1 in the definitive hosts and the environment or of its pathogenicity to humans, which calls for further research. As well as alveolar echincoccosis, cystic echinococcosis is also prevalent in humans in western China [34]. In another recent ME study in China that examined 140 lesions from patients, 108 were caused by *E. granulosus*, one by *E. intermedius*, and 31 by *E. multilocularis* [35]. Misdiagnosis of *E. granulosus* versus *E. multilocularis* was shown to be common in the cases investigated, and the authors conclude that molecular diagnosis is essential for the confirmation of human echinococcosis in the area [35].

## 6. Conclusions

Molecular tools have had a major role in resolving the taxonomy of *Echinococcus*. As a consequence, a practical and informative terminology has been developed for use in epidemiological studies. Importantly, we now have the tools that can be applied in epidemiological investigations. As discussed, these are proving valuable in elucidating life cycles and transmission patterns in endemic areas. This will be of most significance in areas where multiple cycles of transmission operate and where there is the possibility of mixed infections. In terms of clinical management, molecular tools will increasingly inform and guide public health measures. This will be particularly useful in patients diagnosed with echinococcosis but for which the causative species is not known nor whether infection may be due to more than one species of *Echinococcus*. Genotypic diversity within species can now also be detected and correlated with virulence, thus foreshadowing the development of clinically relevant markers.

## Figures and Tables

**Table 1:** Current taxonomy of *Echinococcus.*

Species	Strain/Genotype	Known Intermediate Hosts	Known Definitive Hosts	Infectivity to Humans	Disease
*Echinococcus granulosus*	Sheep/G1	Sheep (cattle, pigs, camels, goats, macropods)	Dog, fox, dingo, jackal and hyena	Yes	CE
	Tasmanian sheep/G2	Sheep (cattle?)	Dog, fox	Yes	CE
	Buffalo/G3	Buffalo (cattle?)	Dog, fox?	Yes	CE
*Echinococcus equinus*	Horse/G4	Horses and other equines	Dog	Probably not	CE?
*Echinococcus ortleppi*	Cattle/G5	Cattle	Dog	Yes	CE
*Echinococcus canadensis*	Cervids/G8,G10	Cervids	Wolves, dog	Yes	CE
*Echinococcus intermedius*	Camel/Pig/G6/G7	Camels, pigs, sheep	Dog	Yes	CE
*Echinococcus felidis*	Lion, dog	Warthog, (zebra, wildebeest, bushpig, buffalo, various antelope, giraffe Hippopotamus?)	Lion	?	-
*Echinococcus multilocularis*	Some isolate variation	Rodents, domestic and wild pig, dog, monkey, (horse?)	Fox, dog, cat, wolf, racoon-dog, coyote	Yes	AE
*Echinococcus shiquicus*	?	Pika and rodents	Tibetan fox and?	?	?
*Echinococcus vogeli*	None reported	Rodents	Bush dog	Yes	PE
*Echinococcus oligarthra*	None reported	Rodents	Wild felids	Yes	PE

Updated from References [2,3]. CE = Cystic Echinococcosis, AE = Alveolar Echinococcosis, PE = Polysystic Echinococcosis. ?: Not known.
